# Effects of masculine culture on the mental health of Northern Sotho male youth

**DOI:** 10.1186/s40359-025-02934-3

**Published:** 2025-06-04

**Authors:** Ntsobe Tshepo Hope Mogano, Daniel Lesiba Letsoalo, Choja Akpovire Oduaran

**Affiliations:** 1 Department of Psychology, Faculty of Health Sciences, School of Psycho-Social Health, North-West University, Mahikeng, South Africa; 2 Department of Psychology, College of Human Sciences, University of South Africa, Pretoria, South Africa

**Keywords:** Mental health, Hegemonic masculinity, Masculine culture, Male youth, Gender norms, Cultural practices, Qualitative, Exploratory

## Abstract

**Background:**

Mental health challenges have become a pressing public health concern in recent years, with the World Health Organization (WHO) reporting a notable prevalence of such difficulties in Africa. Within the region, the contributing factors to this conundrum include inadequate infrastructure, severe underfunding and a strong adherence to masculine culture. However, there is a lack of studies exploring the effects of masculine culture on the mental health of males, particularly Black African males.

**Objective:**

The current study aimed to explore the effects of masculine culture on the mental health of Northern Sotho male youth.

**Methods:**

The study was grounded in a qualitative research approach, an interpretive research paradigm and an exploratory research design. A purposive sampling technique was used to select the sample. Data was collected through individual face-to-face semi-structured interviews and analyzed using qualitative content analysis through the lens of gender role conflict theory.

**Results:**

The findings revealed that the participants’ understanding of masculine culture was centred on the intersectionality of social processes and cultural ideologies. Furthermore, the study revealed that Northern Sotho male youth value masculine ethos; however, this can have negative consequences as failure to meet these expectations may lead to gender role conflict (GRC) and consequently mental health difficulties. Additionally, the study also discovered that in trying to cope with the mental health difficulties, maladaptive mechanisms were adopted. Future studies should include Black male youth from other ethnic groups for a more comprehensive understanding and tracking of the long-term intersectional trajectory of masculine culture and mental health difficulties among men.

**Conclusion:**

The current study provided valuable insight regarding the effects of masculine culture on the mental health of Northern Sotho male youth and underscored the need for culturally sensitive, context-specific awareness campaigns, and targeted interventions to raise awareness and help educate males on the long-term negative implications that masculine culture may have on their mental health.

**Supplementary Information:**

The online version contains supplementary material available at 10.1186/s40359-025-02934-3.

## Background

Increased attention has been drawn to the critical issue of mental health among men worldwide. Societal expectations and stigma often discourage men from seeking help for mental health challenges, resulting in a significant number of untreated conditions. Globally, it is estimated that approximately 970 million people live with a mental disorder, with anxiety and depression being the most prevalent [86]. Mental health issues are particularly pressing in Africa, where access to care remains a significant challenge.

In sub-Saharan Africa, the frequency of mental health problems among men is alarming and varies across regions. The male suicide rate in sub-Saharan Africa stands at 18 per 100,000, which is considerably higher than the global average of 12.4 per 100,000 [32]. The situation is equally concerning in South Africa, where men face disproportionately high rates of mental health difficulties and related deaths. In 2019, approximately 79% of the 13,774 mental health-related deaths in South Africa were men, amounting to a total of 10,861 deaths [73].

These high prevalence rates are worsened by various factors, including poverty, the high prevalence of HIV/AIDS and limited access to mental health services for men [38]. This is particularly troubling because mental health is a critical component of an individual’s overall health and wellbeing [29]. Mental health shapes how individuals feel, think and interact with others [58], as well as how they handle stress and make decisions.

The World Health Organization (WHO) defines mental health as "a state of well-being in which the individual realises his or her abilities, can cope with the normal stresses of life, work productively and fruitfully, and contribute to his or her community ([84](p1)). This definition highlights that mental health is not merely the absence of mental disorders. Instead, it encompasses a broader spectrum of wellbeing, which includes the ability to realise one's potential, manage stress effectively, and positively contribute to one’s community [84].

Mental health prioritization in Africa has been identified as a critical concern. Researchers note that the lack of adequate attention to mental health leads to individuals developing debilitating disorders with significant economic implications. Globally, the World Economic Forum estimated the cost of mental health issues at approximately $2.5 trillion, projected to rise to an alarming $6 trillion by 2030 [28]. These challenges are particularly pronounced in Africa, where competing interests, severe underfunding and inadequate infrastructure exacerbate the situation. In 2022, WHO [85] reported that 116 million people in the African region were living with mental health conditions, a figure higher than the global average. In South Africa, sociocultural factors further complicate the issue, making certain groups, especially men [10], more vulnerable to mental health challenges due to the multicultural context.

Before addressing the interplay between masculinity and mental health, it is important to understand the general state of mental health in South Africa. Socioeconomic inequalities, historical trauma, sociopolitical history, and insufficient mental health services contribute to the prevalence of mental health disorders, making them a significant public health concern. Studies suggest that nearly one in three people in South Africa will face a mental health issue during their lifetime [30]. For example, the South African Stress and Health study reported lifetime prevalence rates of 15.8% for anxiety disorders, 9.8% for mood disorders and 13.3% for substance use disorders [5]. Despite these troubling statistics, only 5% of the national health budget is allocated to mental health services, leaving them severely under-resourced [19]. Men, in particular, face additional barriers to accessing care due to stigma, a shortage of mental health professionals, cultural beliefs and the limited integration of mental health services into primary care systems [19].

Masculinity and masculine culture are shaped by societal norms and expectations, which typically emphasize strength, stoicism and dominance."Hegemonic masculinity", a concept defined by Connell and Messerschmidt [17], refers to culturally endorsed norms that reinforce male power and subjugate women. Adherence to these traditional ideals can result in internal conflict, leading to stress, psychological strain and gender role conflict as men attempt to conform to rigid expectations [59]. Research also highlights the diverse nature of masculinities, and the contradictory pressures men face in their personal and social contexts [83]. Addressing men’s mental health requires an understanding of these dynamics, as strict adherence to traditional masculine norms can have detrimental effects on emotional and psychological wellbeing.

In the South African context, masculinity is closely intertwined with patriarchy, as many indigenous groups remain hierarchically structured, with men positioned at the top. This patriarchal framework often leads to mental health challenges among men being disregarded or trivialized, as societal expectations demand that men remain strong and dominant [14, 37, 67]. Signs of vulnerability, such as expressing emotions or asking for help, are discouraged among males [55], which poses significant risks to their overall quality of life and mental wellbeing. In alignment with traditional values, masculinity in South Africa is frequently linked to heterosexuality and procreation. Men are often expected to fulfil roles as protectors and providers, with fatherhood serving as a critical marker of identity and social status. For many men, fatherhood is not merely a personal achievement but a societal expectation that reinforces their position within their families and communities [47]. These cultural norms create considerable pressure to conform to traditional gender roles, with fatherhood and marriage viewed as essential to achieving recognition, respect and identity [70]. The connection between masculinity, heterosexuality and procreation underscores the importance of family lineage and continuity in many African cultures [41].

In masculine cultures, men who fail to meet societal and cultural expectations often face bashing, humiliation, ridicule and labels such as “weak”. This treatment is particularly evident among men who are perceived as “feminine” or overly connected to women. In many African societies, men considered too “feminine” experience widespread stigma and discrimination. Traditional views of masculinity emphasize strength, dominance and emotional restraint, fostering negative perceptions of men who do not conform [53]. Such individuals are often seen as lacking the traits deemed necessary for leadership and respect within their communities [87]. Consequently, they may endure social isolation, bullying, or even acts of violence [61]. The expectation for men to consistently embody strength is therefore highly oppressive, especially for those unable to meet these rigid ideals of manhood.

For those who strongly adhere to masculine ideology, failure to fulfil their “manly” responsibilities can result in feelings of emasculation. This inability to meet societal expectations often intensifies psychological suffering, leading to pronounced mental health challenges [12, 22, 66]. Men frequently associate masculinity with physical strength, which is linked to protecting the vulnerable and providing leadership [3, 4, 26, 43]. When they fail to fulfil these roles or suppress their vulnerabilities, it can lead to significant mental health consequences. For instance, men may avoid seeking psychological help because of the perception that only “weak” individuals pursue such assistance. An excessively rigid masculine identity can be detrimental to mental wellbeing, contributing to heightened anxiety, depression and feelings of loneliness. This pressure to conform often discourages men from seeking help for mental health issues, as doing so is seen as a sign of weakness [8, 71]. Men in these situations may turn to risky behaviours, substance abuse, or even suicide [71], further exacerbating the harm caused by the suppression of emotions and avoidance of vulnerability [31].

The negative effects of traditional masculine norms are particularly evident within African and South African contexts. Masculinity in these settings is closely tied to cultural expectations and gender roles, which prescribe specific behaviours for men and women. Social norms and mandated traits establish a model of what it means to be a “real man” [78]. Masculine culture forms the foundation of masculinity ideology, characterized by expectations for men to be assertive, tough, competitive, proactive and focused on material success. On an individual level, masculinity is expressed through how one perceives, embodies and performs the concept of manhood [63]. These expectations, however, often have unintended negative consequences for mental health, creating a complex issue which is what this study aimed to address.

### Brief history of Northern Sotho culture

The Northern Sotho people, commonly known as the *Bapedi*people, are one of the indigenous groups found largely in northern South Africa, notably in the Limpopo province [65]. According to several studies, the Northern Sotho culture has been moulded by its history, language, social organisation and traditional customs [35, 51, 64]. The ethnic group speaks “*Sepedi*”, one of South Africa’s 12 official languages. Sign language is now officially recognised as the 12 th language [65].

Cultural traditions, social institutions and historical practices all influence the social organisation of the family among the Northern Sotho people. This is supported by Phillips [62], who found that gender roles play a key role in arranging family life in the Northern Sotho ethnic group. She went on to point out that traditionally, males were responsible for tasks such as hunting, herding and protection, while women were frequently in charge of home administration, childcare and agricultural pursuits. This was and continues to be reinforced by the patriarchal social system, which Nkosi [57] and Palmer and colleagues [60] verified to be a frequent characteristic in Northern Sotho households since the father or eldest male is often viewed as the head of the home. The father has the power to oversee family-related choices and/or decisions. According to Palmer and colleagues [60], patriarchal characteristics are frequently taught and reinforced to young boys during cultural ceremonies, such as traditional male circumcision, also known as “*koma*”, which is not only a rite of passage but also one of the key cultural practices marking the passage from childhood to adulthood for Northern Sotho males.

Mautjana and Makombe [42] alluded that the Limpopo province (one of the provinces in South Africa), where most northern territories are situated, is one of South Africa’s poorest provinces, and while unemployment is a concern throughout South Africa, it is especially prevalent in the Limpopo province. According to Nengovhela [56], the province has the highest unemployment rate and lowest income levels. Statistics South Africa (Stats SA) [74] reported the unemployment rate in the Limpopo province for individuals between the ages of 15 and 64 was 49.5% in 2021. As a result, a study by Mbandlwa and Dorasamy [44] revealed that loss of hope in the province due to unemployment and socioeconomic constraints, which were worsened by the coronavirus disease (COVID-19), among males who conform to the masculine ideology led to psychological distress, as they felt that they were not “man enough” since they could no longer provide for their families – a role that is considered the responsibility of men in the Northern Sotho culture. The context provided above pointed to a need for an investigation into the influence of masculine culture on the mental health of Black South African male youth. As a result, the researchers conducted this study among Northern Sotho male youth in Ga-Mothapo, under the Polokwane municipality of the Capricorn district in the Limpopo province, South Africa.

### Purpose of the study

#### Overarching research question

What are the effects of masculine culture on the mental health of Northern Sotho male youth?

#### Aim of the study

This study aimed to explore the effects of masculine culture on the mental health of Northern Sotho male youth.

#### Objectives of the study

- To establish an understanding of masculine culture among Northern Sotho male youth
- To explore the effects of masculine culture on the mental health of Northern Sotho male youth
- To understand how Northern Sotho male youth deal with mental health difficulties caused by adherence to masculine culture

## Methods

### Study design

This study was grounded in an interpretive research paradigm as it values the understanding and interpretation of the meanings individuals and social groups assign to their experiences, behaviours and the world around them [7]. Through this paradigm, the researchers were able to investigate and comprehend the phenomenon under inquiry by interpreting the meaning that Northern Sotho male youth ascribed to their experiences while considering the importance of context and culture regarding masculinity. The study also adopted a qualitative approach and an exploratory research design. Aspers and Corte [9] indicated that qualitative research gathers and analyses nonnumerical information while focusing on the relevance of the participants’ perspectives, experiences and perceptions of a specific event. This method afforded the researchers the platform to gain a nuanced understanding of how masculine culture affected the mental health of the participants from their perspective. The exploratory research design allowed the researchers to explore in depth the meanings and understanding of the respondents [6]. As a result, researchers could unearth more valuable insights as the topic or research interest has been under-investigated.

### Study area

Participants were recruited from Ga-Mothapo in the Polokwane municipality of the Capricorn district in the Limpopo province, South Africa.

### Sampling technique

The researchers used a nonprobability sampling technique, namely, purposive sampling, to select the sample. Purposive sampling can be described as a sampling technique whereby participants are chosen based on the characteristics that the researcher is interested in exploring [13]. This sampling technique was considered appropriate since the research explicitly focused on a certain group that had to possess certain features, as per the inclusion criteria. These included the following: Participants had to be Northern Sotho males within the age range of youth (15–35 years) as specified by the National Youth Policy (NYP) (2009–2014); be fluent in English or Northern Sotho; reside in Ga-Mothapo in the Polokwane municipality of the Capricorn district, Limpopo province, South Africa; be able to provide consent; and be willing to participate. Recruitment was conducted through distribution of posters during traditional council meetings, local shops, public notice boards at schools and churches, and digitally through various social media platforms.

### Sampling size

The sample size was determined through the principle of data saturation. According to Naeem and colleagues, data saturation in qualitative research signifies “a point whereby data collection and analysis have been exhaustively examined and comprehended, and no additional themes are emerging”. ([54] p1) Data saturation was reached after 13 interviews, leading to a final sample size of 13 participants.

### Data collection procedures

The data were collected through individual face-to-face semi-structured interviews developed by the researchers for this study (see Appendix A). The interviews were recorded, with participants'consent, for later transcription and analysis. The interviews typically took between 40 and 45 min each. English and Northern Sotho were used in the interviews, with the latter being the participants'native language. To ensure the accuracy of the translations, avoid word misinterpretations and incorrect substitution of words, a bilingual Northern Sotho language specialist provided independent assistance in translating the interviews conducted in Northern Sotho into English and then back-translating them. To verify data, minimise bias, enhance credibility and ensure the dependability of the study results, the researchers utilised techniques such as member checking, bracketing, field notes, thick descriptions, reflexivity and journaling.

### Data analysis procedure

The researchers analysed data using seven steps of qualitative content analysis (QCA), namely, (a) preparation of the data, (b) defining the unit of analysis, (c) developing categories and a coding scheme, (d) testing the coding scheme on a sample of text, (e) coding all the text, (f) assessing the coding consistency, and (g) reporting the methods and findings. An independent co-coder conducted a separate analysis to enhance the credibility of the results [36]. After extensive discussion between the researchers and the independent co-coder, only the agreed-upon themes and subthemes made it to the final list. All disagreements were addressed and resolved through consensus within the team being mindful of what the study intended to accomplish. The researchers adhered to the TACT (trustworthiness, auditability, credibility and transferability) framework's principles to ensure the study's robustness and trustworthiness [20].

### Ethical considerations

Before participating in the study, the researchers provided each participant with an information leaflet that explained what the study was about based on their language of preference (either English or Northern Sotho). After this, the participants were provided with a consent form also based on their language of preference, which they had to read and sign as an indication that they voluntarily agree to participate in the study. All the questions that the participants raised were answered honestly by the researchers, and all the important ethical aspects were fully explained to the participants. Participants were also informed that should they feel uncomfortable at any stage, they had the right to withdraw without any consequences. Ethical approval was obtained from the North-West University Health Research Ethics Committee (NWU-HREC) with the ethics number NWU-00009–23-A1. The study was conducted in accordance with the South African National Department of Health Research Ethics Guidelines and the Declaration of Helsinki.

## Results

### Participants

The sample consisted of 13 male participants (n = 13) selected using purposive sampling. All participants were males, aged between 19 and 33 years, 5 were unemployed, 3 were casual workers, 2 were employed and 3 were scholars. A total of 12 participants were single and only 1 was married. In terms of highest level of education, 1 had Grade 10, 1 had Grade 11, 8 had Grade 12, 1 had security Grade E and 2 had tertiary qualifications.

### Findings

The themes and subthemes presented in this section emerged from the QCA data analysis process. The researchers and the independent co-coder had robust discussions and agreed upon the themes and subthemes. The themes and subthemes discussed in this paper are presented in the table below (Table 1). To uphold anonymity, privacy and confidentiality, participants’ real names are substituted with pseudonyms, designated as participants 1 to 13.

**Table 1:** Emergent themes and subthemes

Main themes	Subthemes
Theme 1: Masculinity is a sociocultural concept	Subtheme 1.1: Having a family, being domineering and in control are considered markers of “real men” Subtheme 1.2: Men serve as leaders and mediators within their families and communities Subtheme 1.3: The role of initiation in reinforcing masculine ideologies
Theme 2: Pressure to adhere to sociocultural expectations of being a “real man”	Subtheme 2.1: Being a provider as a marker of manhood Subtheme 2.2: Consequences of failing to meet traditional expectations
Theme 3: Effects of masculine culture on the mental health of Northern Sotho male youth	Subtheme 3.1: Influence of unemployment on masculine identity and mental health Subtheme 3.2: Perception of work and its role in masculine status
Theme 4: Masculine culture serves as a barrier to seeking help and promotes self-reliance	Subtheme 4.1: Perceptions of weakness associated with seeking help
Theme 5: Coping mechanisms used to address mental health difficulties	Subtheme 5.1: Maladaptive coping through substance use Subtheme 5.2: Emotional withdrawal and avoidance as coping strategies

### Theme 1: Masculinity is a sociocultural concept

This theme reflects participants'understanding of masculinity as shaped by social processes and cultural ideologies. Masculinity was described as encompassing how men should handle themselves and fulfil roles, such as being in control, employed and providing for their families. Participant 5's response highlights this: “…*I would say masculine culture has to do with how men are supposed to handle themselves in different areas of their lives (command the respect of others and not act like a small child; he must be in control of his life, have a job and provide for his family)…”.*

Participants highlighted that masculine culture includes traditional teachings from elders, emphasizing stoicism, leadership and protection of women. The latter point is illustrated in participant 7's response: “…*I think that it is how men are taught to live and how they should handle themselves (not to cry or panic when he is in trouble or show fear and that we as men are heads and protectors of women). Something like traditional rules from elders. I mean, it’s like the rules that we get from our elders as men; it’s like directions in a way for men. You know, how to live your life as a man…”.* These teachings are passed down through mentorship by men within families and communities to preserve and uphold masculine expectations across generations. The following excerpt from participant 5 affirm this: “…*I think it’s something that you learn from other men. It’s like teachings from older men…as in men who came before you. Your father, your uncles and maybe grandfather…”.* Overall, masculinity is understood as a construct deeply rooted in societal and cultural norms.

### Subtheme 1.1: Having a family, being domineering and in control are considered markers of “real men”

This subtheme highlights participants’ views on what defines a “real man”. Many believed that being a “real man” involves having a family, being in control, and commanding respect both within the household and the community. Participant 3's response demonstrates the latter assertion: “…*well, a man must have a family of his own, he must be able to control his family, do you understand me? When he speaks, his family must listen to him…”* Likewise, men are expected to handle themselves with authority, serve as the head of the family, and lead their families toward prosperity through vision and decision-making. The latter is comprehensively captured in participant 5's response: “…*I would say a man is supposed to handle himself in a certain way in different areas of his life. In his family and the community. I mean in his home; his family must recognise that he is the head of the family, and they must listen to him, and in his community, he must also be respected…”.*

Respect and dominance were viewed as essential qualities, reinforcing the notion that a “real man” must have vision and provide for his family and fulfil traditional masculine roles. The latter assertion is confirmed by an excerpt from participant 8: “…*I believe as a man you must have a vision and make decisions. Only real men with a vision can lead their families to greener pastures. His family can prosper and not go hungry. Many families fail to reach such prosperous lands because the man does not have a vision, or his wife and children may lack control…”.*

For participants, having a family symbolised not only manliness but also the continuation of the ethnic group and societal expectations. An excerpt from participants 3 illustrate this: “…*when they say “o monna gare ga banna” (you are a man among men), it means that you are respected amongst other men. As I said before, a man must command respect from others, and I want to be respected in my community and my family one day…”.*

Ultimately, “real men” are characterized by having a family, being dominant, in control and acting as providers, leaders and authority figures in their families and communities.

### Subtheme 1.2: Men serve as leaders and mediators within their families and communities

This subtheme highlights participants’ views on the role of men as leaders and mediators within their families and communities. Participants emphasized that being a “real man” involves taking initiative and resolving household conflicts in ways that demonstrate leadership and avoid physical confrontation. This view is demonstrated in the response provided by participant 1: *“…a man is somebody who takes initiative at home. It is someone who will resolve conflict when it arises in his household…”* Likewise, participant 13 concurred and illuminated “*…as in the family, maybe there is a conflict of some sort; his [a man] decisions on how to handle the matter will show that he is a real man. As a real man, he must be able to solve any conflict in the household without showing physical strength or fighting. He must be a leader in the community and his family and help others in the community…”.*

Leadership extends beyond the household to the community, where men are expected to help others and maintain respect. The expression by participant 6 encapsulates this: “…*men are leaders of the community and leaders of their households, and as such, I think it is important for men to be viewed as leaders. A leader who will offer leadership in his community and lead his family…”* Eventually, participants agreed that a man’s “manliness” is defined by how others perceive his ability to lead and mediate effectively in both familial and communal contexts.

### Subtheme 1.3: The role of initiation in reinforcing masculine ideologies

This subtheme highlights how cultural customs, rituals and rites of passage, such as traditional male circumcision (“*koma*” in Northern Sotho), solidify one’s “manliness”. Participants emphasized that initiation school serves as a critical rite of passage where boys learn masculine teachings and gain recognition as “real men”. Participant 1’s response illustrates the latter: “…*I believe that a man is a real man after he has come back from the mountains. Therefore, for a man to become a real man, he must go to the mountains because only then will he learn what other men are being taught in the mountains…”.*

Despite some expressing pressure to undergo initiation, they endorsed its importance for acquiring respect and avoiding ridicule within their communities. Participants 4 endorsed this view and illuminated: *“…I have heard a lot of boys feeling pressure to go to the mountains, but I feel like as a man you need to go to the mountains, that is where you learn about being a man…”* Participant 7 concurred and stated: “…*Yeah. I mean, I went to the mountains under the pressure of wanting to be a “real man”. I used to get teased a lot because most of my friends went to the mountains and I didn’t…”* For those who do not attend, the social stigma can lead to emotional distress and potential mental health challenges. Initiation is therefore viewed not only as a cultural tradition but as a method to instil and reinforce masculine ideologies across generations.

### Theme 2: Pressure to adhere to sociocultural expectations of being a “real man”

This theme highlights how men must adhere to traditional expectations, particularly serving as the primary breadwinner, in order to establish their status as “real men” within their community. This requires securing employment and contributing financially to their household and community, with failure to do so often resulting in immense pressure and mental health challenges. Participants expressed that being a provider affords men respect, dignity and recognition as leaders and “men among men”. However, failing to meet these expectations leads to strain, lack of respect and susceptibility to mental health difficulties. This theme will be further substantiated by the subtheme presented below.

### Subtheme 2.1: Being a provider as a marker of manhood

This subtheme arose from participants stressing men's role as main providers in their families and communities. The focus is on the societal pressure on men to get jobs, provide financially for their families, and thus prove their masculinity. Providing is described by participants as vital for self-worth and adherence to cultural responsibilities. The following quote from participant 1 who was unemployed during data collection illustrated this: “…*I believe if I can get employment and be able to contribute to my family and be able to contribute and help the community. I would like to be able to provide for my children one day so that even the community can see that I am a man because I am a provider…”.*

Participant 13 agreed and illuminated: *“…I believe that every man must be able to provide, he must have a job so that he can provide for his family and help the community so that he can show that he is a man. When a man doesn’t have a job, his manliness doesn’t show because he won’t be able to do anything for himself or his family. Therefore, it is important that he gets a job and has his things to show his manliness…”* It is evident that financial success is linked to ideas of masculinity, suggesting that not meeting these expectations can cause men to feel inadequate and socially unacceptable.

### Subtheme 2.2: Consequences of failing to meet traditional expectations

This subtheme emerged from the participants views which reflected the psychological strain and mental health difficulties experienced by men who are unable to fulfil their culturally and socially defined roles as providers. Men face significant emotional and psychological pressure due to their inability to conform to traditional ideals of masculinity. Participants illustrate how this failure can result in societal rejection, diminished respect and a sense of emasculation, which in turn impacts their mental wellbeing and increases susceptibility to mental health challenges. A response from participant 3 demonstrates this: “…*you attain that respect by respecting yourself and not letting your family down. You must be able to provide for your family and do everything you can to make sure that your family is well taken care of…”.*

### Theme 3: Effects of masculine culture on the mental health of Northern Sotho male youth

This theme examines the effects of masculine culture on the mental health of Northern Sotho male youth. Participants revealed that failing to meet masculine expectations left them feeling embarrassed, worthless and unworthy of respect. Unemployment further contributed to feelings of hopelessness and isolation, as men struggled with their inability to participate in activities that affirmed their manhood. Additionally, the type of job a man holds plays a significant role in how he is perceived, with less respected jobs causing discouragement and social ridicule, leading some to withdraw or seek work far from home. Men’s inability to conform to traditional norms and expectations results in strain, manifesting as discomfort, withdrawal, hopelessness, fear and worry about their future and societal roles. This theme will be elaborated through the subthemes presented below.

### Subtheme 3.1: Influence of unemployment on masculine identity and mental health

This subtheme arose from participants'descriptions of how failing to meet traditional masculine ideals (steady employment, financial stability) diminishes their self-respect and self-worth. Participant 1 illuminated: “…*I feel unworthy of respect…”* Participant 9 concurred and stated: “…*most of the time, I cannot afford, and they would offer to buy me. It is embarrassing being dependent on another as a man*…” Participant 10 agreed and decried: “…*Yeah, sometimes you will feel like you are nothing…”.*

Unemployment also led participants to feel embarrassed, worthless and unworthy, hindering their involvement in traditionally masculine social activities. Participant 7’s response captures this: *“…I mean sometimes I cannot even participate in activities that other men are doing, I tend to feel less about myself…”* Participant 13 echoed similar sentiments and stated: *“…sometimes it is truly, truly, heavy, and you start asking yourself if your situation will ever be better, and you start losing hope…”* The absence of fulfilment resulted in isolation and hopelessness, evident in expressions of the emotional burden of financial dependence.

### Subtheme 3.2: Perception of work and its role in masculine status

This subtheme arose from the participants descriptions of how a man's work shapes his masculine identity and community standing. Participants reported that jobs considered less respectable, like gardening, led to ridicule and lower social status. To validate the latter, participant 6 expressed: *“…I mean who respects a garden boy currently?”* Due to this perception, participants avoided their local community by seeking work elsewhere, to escape judgment. Participant 6 further illuminated: “…*sometimes I do not even feel like going to look for jobs around the community; I just stay in the house the whole day doing nothing. Rather, I look for gardening jobs far away in another community because sometimes I even get ridiculed by schoolchildren who are much younger than me. Sometimes I just want to beat them, but I’m afraid of their parents, so when I get truly angry, I threaten and swear at them when they disrespect me…”* The stress and shame intensified their pre-existing mental health problems, triggering a range of emotions from anger to anxiety about their social standing and future.

### Theme 4: Masculine culture serves as a barrier to seeking help and promotes self-reliance

This theme reflects participants’ views on men seeking help, highlighting that many avoid it due to the belief that it signals an inability to handle their problems. Participants expressed that seeking help is seen as a weakness, potentially exposing men to judgment, ridicule, or attacks. This perception drives a preference for self-reliance, as men strive to uphold the masculine ethos of independence and strength. However, this avoidance of external assistance often results in bottling up issues, leading to negative long-term mental health consequences. This theme will be elaborated on through the subthemes below.

### Subtheme 4.1: Perceptions of weakness associated with seeking help

This subtheme highlights participants'perception of seeking help as a sign of incapability or weakness. Participants worried that seeking help might make them seem weak or incompetent, potentially inviting criticism and ridicule. To support this, participant 6 stated: “…*I don’t like asking for help, it’s like crying for help, it makes you look like you can’t handle your problems. I handle it by myself…”.*

They highlighted the societal expectation of self-reliance and strength, discouraging vulnerability to avoid judgment or criticism. This belief is ingrained in traditional masculinity, which avoids vulnerability and thus resists outside assistance. Participant 11 response captures this: *“…Ah! I always try to solve my problems by myself. I don’t like involving people in my problems or things I am struggling with. I don’t like people knowing that I am struggling and next you know they will judge and think that you can’t face your problems without others…”* Participant 13 concurred and stated:* “…Ah, you know how life is. As a man, like I said you must avoid presenting yourself as weak to the world. That’s how people attack you. They will make you feel like you are nothing and you can’t do anything, next thing you find the entire community laughing at you. Therefore, I stand by myself in all situations…”.*

### Theme 5: Coping mechanisms used to address mental health difficulties

This theme highlights how participants cope with mental health difficulties caused by failing to meet masculine expectations, with most strategies being maladaptive. Some participants turned to drinking to escape their frustrations and reduce stress. Others coped through emotional withdrawal, such as prolonged sleep, to avoid confronting feelings of hopelessness and worthlessness. Participants expressed a reluctance to seek external help, fearing it would make them appear “weak”. As a result, their reliance on maladaptive strategies often exacerbates their challenges. This theme will be elaborated through the subthemes presented below.

### Subtheme 5.1: Maladaptive coping through substance use

This subtheme highlights how some participants used alcohol to cope with the mental health challenges of not living up to masculine ideals. Participants stated alcohol provided temporary relief from stress and frustrations, enabling relaxation. Participant 11 illuminated: *“…I am talking from experience. When I feel like there are too many problems, I drink at the tavern; it helps me relax and stop stressing…”* Participant 5 concurred and expressed: *“…I mean, I could say that I may also have drank (alcohol) a lot because of my frustrations and so did many other guys out there…”* However, this coping strategy shows a reluctance to seek help externally, fuelled by a fear of seeming weak.

### Subtheme 5.2: Emotional withdrawal and avoidance as coping strategies

This subtheme highlights how other participants managed mental health challenges by withdrawing emotionally and physically. Participants described escaping feelings of worthlessness, hopelessness and worry by sleeping excessively. Participant 6 stated: *“…Sometimes I would sleep the entire day thinking that I would never be like other men who have jobs and families…”* These non-aggressive actions reveal a hidden fight against societal disapproval. This subtheme highlights the negative impact of rigid masculine expectations, which cause emotional distress and prevent men from seeking help.

## Discussion

The findings of this study suggest that participants’ conceptualisation of masculine culture is centred on socialisation, gender roles and behavioural expectations making it a sociocultural concept. It appears that the construction of masculinity among young males is a process that involves elderly men who directly or indirectly impart their knowledge and understanding, essentially teaching them how to behave, conduct themselves and uphold their “manliness”. This finding affirms the results of the study by DeCaille [21], who discovered that in most African cultures elderly men and fathers play a significant role in instilling cultural ethos in their sons and boys, either directly by grooming them or indirectly by influencing their conduct in the household and the community. This process is believed to teach their sons how to act and carry themselves publicly to demonstrate their “manliness” to others.

The study also discovered that participants embrace the stance of men being the heads of families, leaders and ultimate decision-makers, attributing family domination and control to what a “real man” is. The participants considered a “real man” to be someone who is able to lead and manage the family without opposition from other members. Most participants agreed with this attitude, equating family dominance and control to what a “real man” is. This finding is consistent with the results of several African studies which all confirmed that fathers are usually the major decision-makers in African families [1, 23, 25, 33, 75]. The latter responses also appear to endorse the Northern Sotho proverb of “*Monna ke hlogo ya lapa*,” (a man is the head of the family) as alluded to by Selepe, who argued that such proverbs strengthen a man’s role as the final decision-maker in all important household matters [69]. Although not openly voiced by the participants, it appears that the participants’ conceptualisation of masculinity closely resembles what Connell termed “hegemonic masculinity” [16].

Most of the participants in this study believed that for a man to exhibit his “manliness”, he must have his own family. This implies that getting married, having a wife and children are some of the most important achievements in a man’s life – a belief tied to the principle of procreation. That is, a man’s ability to procreate, start and maintain a family is one of the requirements for the recognition of one’s “manliness”. This affirms findings by Selepe [69], who discovered that among the Northern Sotho’s, the best way to demonstrate masculinity is by means of procreation and fathering. Even though having children appears to be a vital role for males and a sign of “manliness”, according to the participants, fathering extends beyond recognition to preserving the family, managing the home and actively fathering the children, as it is through active involvement that sons will be socialised and adequately prepared to take on active responsibilities of being the primary breadwinners of their families in the future [68]. The latter is not surprising, as most indigenous groups in Africa do not approve of men not having children. Failure to uphold this expectation of procreation can have detrimental consequences. For example, in Ghana, males who do not procreate are not only considered “not man enough”, but they are also socially ridiculed, scorned, treated inhumanly, buried on isolated land, and their genitals pierced after death to indicate that their sexual organs were not used to procreate [34]. This demonstrate that men who are infertile within such communities will feel emasculated and this will likely lead to mental health challenges. The possibility that some men may be sterile is often overlooked or not considered as there is a blind focus on procreation.

The participants in this study also emphasised the importance of initiation school as both a rite of passage and a means through which masculine ethos are instilled, reinforced and maintained. Traditional circumcision, referred to as “*koma*” in Northern Sotho culture, appears to be a consistent ritual and rite of passage into manhood among most African cultural groups [40]. Within the South African context, this practice is most notable among the Xhosa people [50]. Within the same context, if boys do not go through this ritual, they will be ridiculed, and their manhood will be called into question by their peers leading to gender role conflict [40, 45]. The fear of being emasculated by peers due to being uncircumcised is a consistent concern, which was also noted among young men in Nairobi [40]. This can also lead to unintended mental health difficulties.

The participants in this study also emphasised the importance of acquiring employment, as it is regarded as one of the most important factors in attaining the status of being considered a “real man”. Employment is perceived as a vehicle to provide for families as men are usually primary breadwinners. This finding is consistent with the results of various studies which illuminated that traditional masculine ethos require men to be independent or primary breadwinners of their households [11, 25, 27, 46, 48, 52, 68, 72, 81]. Within the same context, others expressed that inability to conform to employment expectations can negatively affect mental health, leading to feelings of shame or inadequacy. These findings imply that, in trying to uphold sociocultural and traditional gender roles and masculine expectations, young males might experience distress or gender role conflict [59]. The expectation puts pressure on men to suppress their emotions, which can lead to negative consequences. The latter is more pronounced in men who are unemployed as they are not respected due to their inability to provide and take care of their families unlike their employed counterparts [77]. That is, a lack of respect for unemployed men, within the context of masculinity, can have a significant impact on their mental wellbeing and social connections. Traditional ideas of what it means to be a man often highlight the value of respect and social standing, and when men feel disrespected, it can cause them serious mental health problems. The distress is associated with feelings of inadequacy and a reduced sense of self-worth, according to Wade and Rochlen [77]. Furthermore, the pressure to conform can increase stress and contribute to unhealthy ways of dealing with it, such as aggression or substance use [49]. Internalising disrespect can lead men to suppress their emotions and avoid vulnerability, further isolating them and hindering their ability to seek support [49]. Solving these issues necessitates a shift away from traditional masculine expectations and the fostering of a culture that values emotional expression and mutual regard.

However, despite the above-mentioned gender role conflict most participants indicated that they would still have to act strong and not seek help. This finding echoes the masculine ideals presented by Cousin and colleagues [18] and Williams [80], who both pinpointed stoicism as one of the ideal masculine traits, suggesting that even in the face of hardship and pain, men are encouraged to endure without showing feelings – a term called gender role constraint. The concept of gender role constraints was well described by White [79], who highlighted how men may feel confined by their gender roles, restricting their own behaviour to conform to stereotypical masculine norms. Some of the views expressed by the participants in this study on vulnerability, as well as the vulnerability of others, reflect the same thoughts. The participants stated that men should not cry because if they do, they will lose respect, and their manhood will be called into question. Participants further stated that only weak boys and women cry. This belief is often perpetuated by masculine expectations and stigma surrounding vulnerability, which are still prevalent among most Africans cultural groups [24]. The latter underpins the assertion by Cole and Ingram [15], who asserted that during masculine indoctrination, males are encouraged to suppress their vulnerable emotions. Similarly, Mabrouk [38] concurred and indicated that vulnerability is viewed as being associated with femininity or weakness. In effect, it is evident that the lack of emotional expression and vulnerability in men, often encouraged by traditional masculine norms, undeniably leads to significant psychological and social effects. Strict adherence to these norms can discourage men from seeking mental health help, contributing to higher rates of untreated mental health conditions such as depression and anxiety [39]. Suppressing emotions can lead to more stress, damaged relationships and a greater chance of turning to substances to cope [2]. Additionally, the pressure to be stoic and self-reliant can isolate people and prevent them from forming deep, supportive friendships, which are essential for emotional health [83]. These contributing factors create a situation where men are more likely to take their own lives compared to women, emphasising the need to alter cultural norms and encourage men to show their feelings and be open.

In addition, endorsing suppression of feelings and discouraging vulnerability, the participants supported the masculine principle of self-reliance as opposed to seeking external assistance. In reaction to the prospect of seeking external help, the participants stated that they encourage self-reliance even during difficult situations, as asking for help is perceived as a sign of weakness. By doing so, the participants perpetuate what O’Neil [59] referred to as gender role violation; this may be towards the self and others by expressing and encouraging self-reliance rather than authenticity and vulnerability. Authenticity and vulnerability were considered to be outside of traditional masculine norms, as their (participants’) response appears to potentially condemn those who may be authentic and vulnerable in times of difficulty. Additionally, those who are too feminine, close to women or strongly adhere to the prescribed masculinity standards and fail to uphold them may be seen as not being “real men” or man enough. As a result, male gender role conflict may arise within these males and manifest as negative affective emotions such as fear, worry and great sadness or low moods. Regarding strategies used by the participants to deal with mental health difficulties, the study discovered that the participants mostly used maladaptive mechanisms such as drinking alcohol, withdrawal and detachment from social contexts.

Overall, findings from the study indicate that men who have difficulty achieving dominant masculinity may suffer from various negative impacts on their mental and social wellbeing. They may face considerable psychological stress due to the pressure to meet societal expectations of masculinity which value traits such as emotional stoicism, physical strength and financial success [39]. This struggle can contribute to feelings of inadequacy and low self-esteem, leading to increased depression and anxiety rates [82]. In addition, men who deviate from these norms may experience social rejection and prejudice, which can amplify their mental health struggles [76]. Men's struggle to reconcile their personal identity with societal expectations can lead to unhealthy coping strategies, like substance abuse and aggression, as they try to assert their masculinity [2]. Resolving these problems requires a cultural change that embraces more inclusive and flexible ideas of masculinity, enabling men to express their vulnerability and seek support without fear of being judged.

### Limitations and recommendations

Although the study yielded useful insights, there are some limitations to be acknowledged. Firstly, the study was restricted to Northern Sotho male youth. Considerably, for an integrated understanding, more research will need to be conducted to include other ethnic groups of male youth, as their views might echo or contradict the ones found in this study as South Africa is multicultural. This study also used individual face-to-face semi-structured interviews to collect data; therefore, further studies could consider adding a quantitative component, focus groups or explore the phenomenon thoroughly using mixed methods. Additionally, the current study used an independent bilingual translator; therefore, there is a possibility that the back-translation might have led to the incorrect substitution of some words. Furthermore, the study used purposive sampling to select the sample; therefore, the results cannot be generalised to the overall population of Northern Sotho male youth. Future studies should consider using probability sampling techniques and a larger sample size to make generalisability feasible. Finally, it must be categorically stated that although the study managed to discover some of the mental health difficulties following intense probing during data collection, the researchers are mindful that the insights discovered are not exhaustive. That is, due to the sensitive nature of the topic, the participants may not have been fully revealing as being too forthcoming may be equated to weakness, and something that is not encouraged in masculine culture. However, despite this limitation, the insights discovered in this study are valuable and should be viewed as a start and a foundation from which future studies on masculinity within the context of Black men can be built upon.

## Conclusion

This study aimed to investigate the effect of masculine culture on the mental wellbeing of Northern Sotho male youth. The study provides valuable insights. One of the key findings is that while adhering to masculine culture is valued, it also has negative consequences. Failure to live up to masculine ideals may lead to gender role conflict, which in turn has negative effects on the mental health of men. Masculine culture also seems to promote self-reliance, discourages seeking help, and therefore predisposes men to engage in maladaptive coping mechanisms. In light of this, further research is needed to better understand the issue broadly. The study's results offer valuable insights for developing context-specific, sensitive, relevant and responsive mental health awareness campaigns and targeted interventions for men where the study was conducted. This will ensure that these programmes are not only sensitive to the needs of the local population but can effectively promote mental health awareness and education regarding the intersectionality between masculine culture and mental health.

## Supplementary Information

Supplementary Material 1.

## Data Availability

The data that supports the findings of this study is available on  reasonable request from the corresponding author.

## Abbreviations

Stats SA: Statistics South Africa
WHO: World Health Organization
WEF: World Economic Forum
NYP: National Youth Policy
GRC: Gender Role Conflict
COVID-19: Coronavirus Disease
QCA: Qualitative Content Analysis
TACT: Trustworthiness, Auditability, Credibility and Transferability
NWU-HREC: North-West University Health Research Ethics Committee
