# Supplementary material for: Effects of masculine culture on the mental health of Northern Sotho male youth
Source: BMC Psychol. 2025 Jun 4;13:605. doi: 10.1186/s40359-025-02934-3 (PMC12139181; doi:10.1186/s40359-025-02934-3)
Supplement: Supplementary file 1 — Supplementary Material 1. [file 40359_2025_2934_MOESM1_ESM.pdf]

Appendix A: Semi-Structured Interview Guide – English version

Section A: Demographics

- Name (pseudonym will be given):
- Age:
- Occupational/Employment status:
- Ethnic group (Northern Sotho only):

Section B: Interview Questions

The researchers utilised the interview questions as a guide and conducted probing whenever necessary to uncover deeper subjective meanings, leveraging the inherent flexibility of semi-structured interviews.

1. May you kindly share with me your understanding of what “masculine culture” is?
   - ✓ ***Probe***: What is your understanding of the term “masculine culture”?
2. Can you share with me in detail your views on what is socially expected of a man?
3. May you share with me the things that you think one would have to do to show that he is a “real” man?
4. Have you ever felt pressured to do what other men are doing? If yes, what is it that you have done?
5. Do you think it is important for you to be viewed as a “man amongst men”? If yes, what does this mean, and what makes this important in your view?
6. What are your views on how a man should express himself in various contexts?
7. What do you consider important to achieve for one to be regarded as a man?
8. Do you ever worry about not coming across as man enough to other men and women? If yes, how do you handle this?

- ✓ ***Probe***: How do you cope when you feel like you are not man enough?

9. How do you feel about crying as a man?

   - ✓ ***Probe***: How would you react if you saw another man crying?
   - ✓ ***Probe***: How would you advise him?

10. How often do you ask for help, and where?

   - ✓ ***Probe***: How often do you consult at a clinic, and would you consult a counsellor or clinical psychologist? If not, why not? Please elaborate.

11. Anything else you would like to share?

Thank you very much for your participation in this study
